# Crystal structures of [(μ_2_-L1)di­bromidodicopper(II)] dibromide and poly[[(μ_2_-L1)diiodido­dicopper(I)]-di-μ-iodido-dicopper(I)], where L1 is 2,5,8,11,14,17-hexa­thia-[9.9](2,6,3,5)-pyrazino­phane

**DOI:** 10.1107/S2056989020007161

**Published:** 2020-06-02

**Authors:** Tokouré Assoumatine, Helen Stoeckli-Evans

**Affiliations:** a Institute of Chemistry, University of Neuchâtel, Av. de Bellevax 51, CH-2000 Neuchâtel, Switzerland; b Institute of Physics, University of Neuchâtel, rue Emile-Argand 11, CH-2000 Neuchâtel, Switzerland

**Keywords:** crystal structure, pyrazino­phane, hexa­thia­pyrazino­phane, copper(II), copper(I), binuclear complex, two-dimensional coordination polymer, supra­molecular network

## Abstract

The reaction of the hexa­thia­pyrazino­phane ligand, 2,5,8,11,14,17-hexa­thia-[9.9](2,6,3,5)-pyrazino­phane (**L1**), with copper(II) dibromide led to the formation of a binuclear complex, [μ_2_-2,5,8,11,14,17-hexa­thia-[9.9](2,6,3,5)-pyrazino­phane]bis­[bromi­docopper(II)] dibromide, [Cu_2_Br_2_(C_16_H_24_N_2_S_6_)]Br_2_, (**I**). The complex possesses inversion symmetry with the pyrazine ring being situated about a center of symmetry. The ligand coordinates to the copper(II) atom in a bis-tetra­dentate manner and the copper atom has a fivefold NS_3_Br coordination environment with a distorted shape. The reaction of ligand **L1** with copper(I) iodide also gave a binuclear complex, which is bridged by a Cu_2_I_2_ unit to form a two-dimensional coordination polymer, poly[[μ_2_-2,5,8,11,14,17-hexa­thia-[9.9](2,6,3,5)-pyrazino­phane]tetra-μ-iodido-tetra­copper(I)], [Cu_4_I_4_(C_16_H_24_N_2_S_6_)]_*n*_, (**II**). The binuclear unit possesses inversion symmetry with the pyrazine ring being located about a center of symmetry. The Cu_2_I_2_ unit is also located about an inversion center. The two independent copper(I) atoms are both fourfold coordinate. That coordinating to the ligand **L1** in a bis-tridentate manner has an NS_2_I coordination environment and an irregular shape, while the second copper(I) atom, where **L1** coordinates in a bis-monodentate manner, has an SI_3_ coordination environment with an almost perfect tetra­hedral geometry. In the crystal of **I**, the cations and Br^−^ anions are linked by a number of C—H⋯S and C—H⋯Br hydrogen bonds, forming a supra­molecular network. In the crystal of **II**, the two-dimensional coordination polymers lie parallel to the *ab* plane and there are no significant inter-layer contacts present.

## Abstract 2

The reaction of the hexa­thia­pyrazino­phane ligand, 2,5,8,11,14,17-hexa­thia-[9.9](2,6,3,5)-pyrazino­phane, with copper(II) dibromide lead to the formation of a binuclear complex. Reaction with copper(I) iodide also gave a binuclear complex, which is bridged by a Cu_2_I_2_ unit to form a two-dimensional coordination polymer.

## Chemical context   

Tetra­substituted pyrazines are inter­esting ligands for the formation of multi-dimensional coordination polymers and metal-organic frameworks: for example, tetra-2-pyridyl­pyrazine (Ouellette *et al.*, 2004 ▸; Nawrot *et al.*, 2015 ▸) and pyrazine­tetra­carb­oxy­lic acid (Masci & Thuéry, 2008 ▸; Zhang *et al.*, 2014 ▸). In recent years a new ligand, 2,3,5,6-(4-carboxyl-tetra­phen­yl)pyrazine, has been used successfully to form a number of metal–organic frameworks (Wang *et al.*, 2019 ▸).

**Figure 1:**
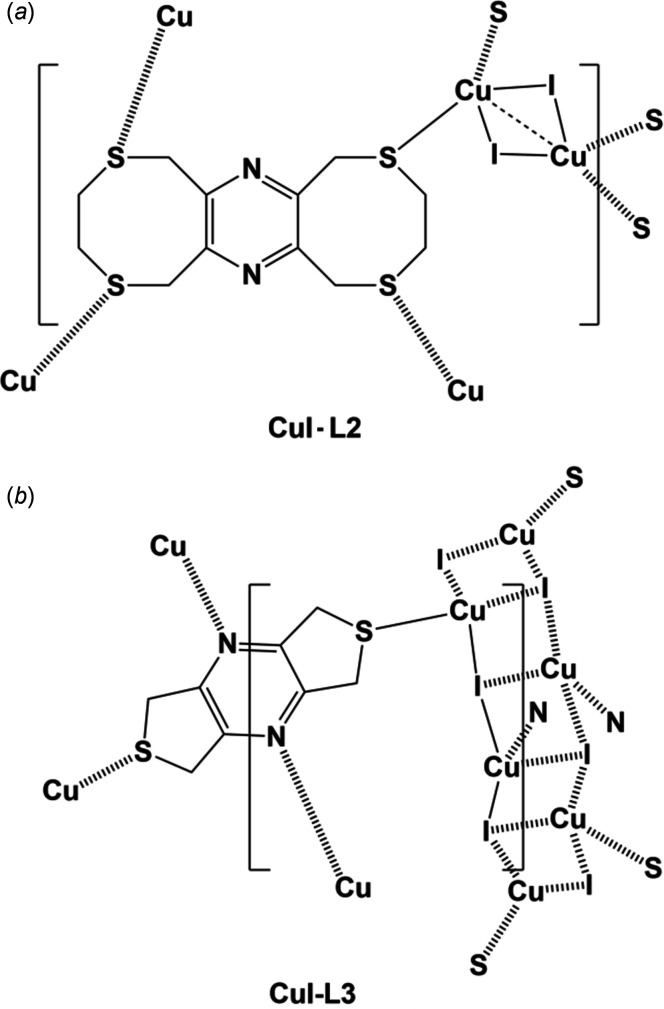
Chemical drawings of the complexes involving CuI and ligands **L2** and **L3**.

A number of such ligands involving N_pyrazine_ and S coordin­ation sites have been synthesized and their coordination behaviour with transition metals investigated (Assoumatine, 1999 ▸). The title ligand, **L1** (Assoumatine & Stoeckli-Evans, 2020*a* ▸), is the third in a series of pyrazine­thio­phane ligands that have been shown to form chains, networks and frameworks with copper halides (Assoumatine, 1999 ▸), especially with CuI. For example, ligand **L2**, 3,4,8,10,11,13-hexa­hydro-1*H*,6*H*-bis­([1,4]di­thio­cino)[6,7-*b*:6′,7′-*e*]pyrazine, when reacted with CuI formed a two-dimensional coordination polymer, poly[[μ_4_-3,4,8,10,11,13-hexa­hydro-1*H*,6*H*-bis­([1,4]di­thio­cino)[6,7-*b*:6′,7′-*e*]pyrazine]­di-iodido-­dicopper(I)] (Fig. 1 ▸ *a*; Assoumatine & Stoeckli-Evans, 2020*b* ▸). Ligand **L3**, 5,7-di­hydro-1*H*,3*H*-dithieno[3,4-*b*:30,40-*e*]pyrazine, when reacted with CuI formed a three-dimensional coordination polymer, poly[(μ_4_-5,7-di­hydro-1*H*,3*H*-dithieno[3,4-*b*:3′,4′-*e*]pyrazine-*κ^4^N:N′:S:S′)*tetra-μ_3_-iodido­tetra­copper] (Fig. 1 ▸ *b*; Assoumatine & Stoeckli-Evans, 2020*c* ▸). Inter­estingly, in compound **CuI-L2** the copper atom does not coordinate to the pyrazine N atom, whereas in compound **CuI-L3** one of the two independent copper atoms does coordinate to the pyrazine N atom. Herein, we report on the results of the reactions of ligand **L1** with CuBr_2_ and CuI, where in both cases the pyrazine N atom is involved in coordination to the copper(II) and copper(I) atoms, respectively.
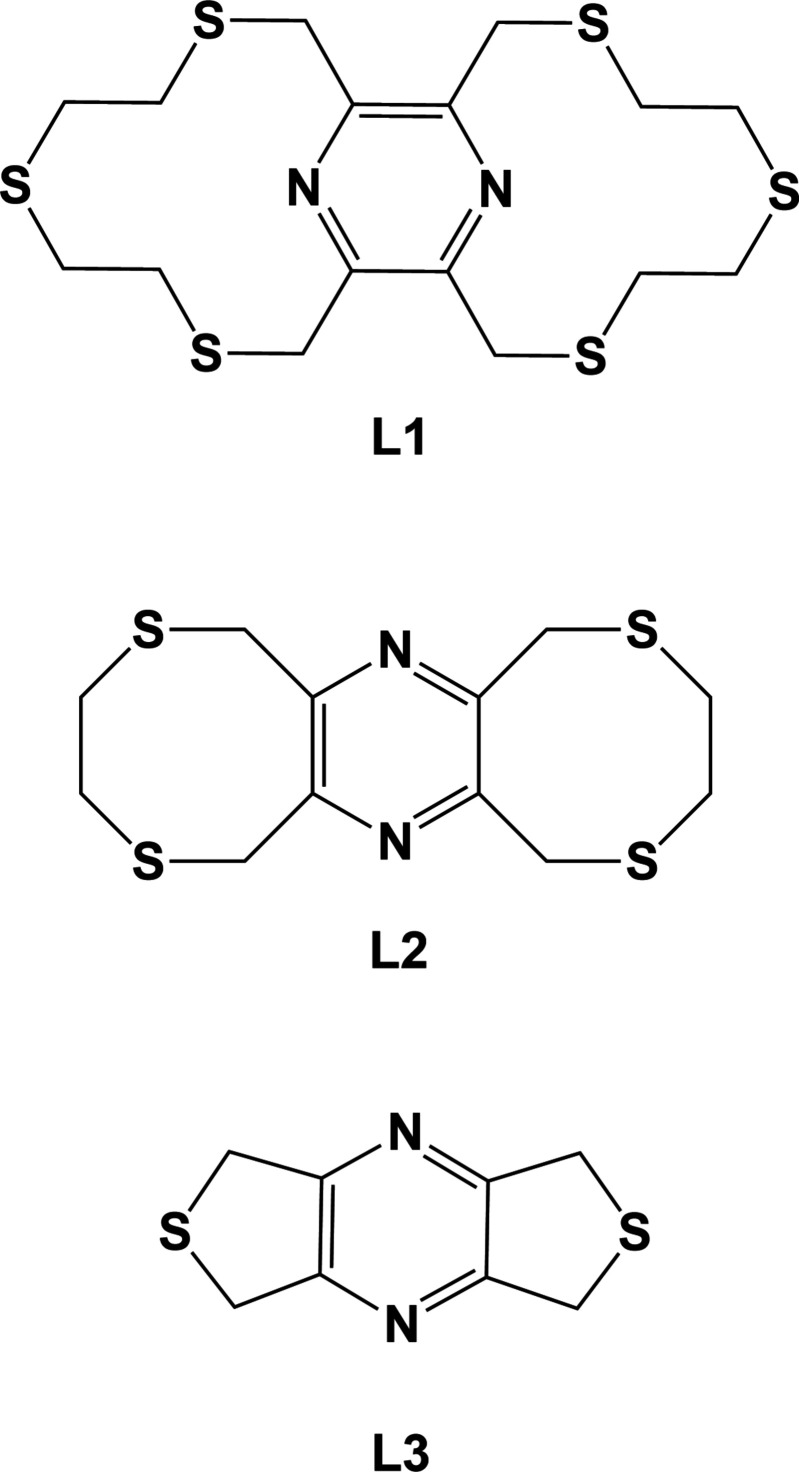
 
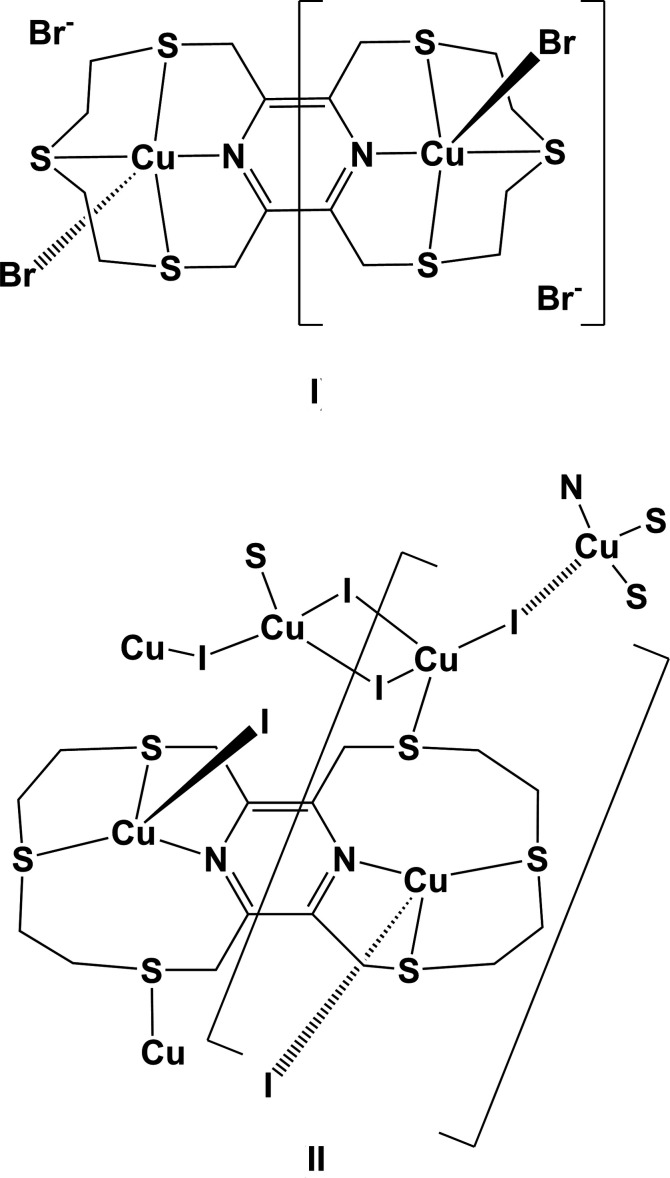


## Structural commentary   

**Figure 2:**
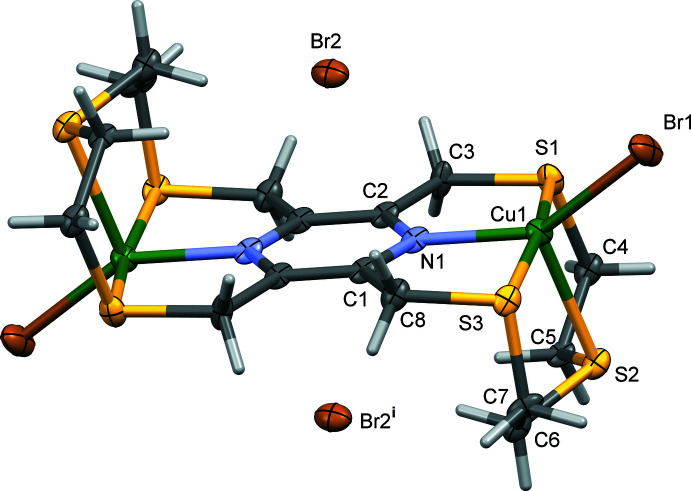
A view of the mol­ecular structure of complex **I**, with atom labelling for the asymmetric unit; symmetry code: (i) −*x* + 1, −*y* + 1, −*z*. Displacement ellipsoids are drawn at the 50% probability level.

The reaction of the hexa­thia­pyrazino­phane ligand, 2,5,8,11,14,17-hexa­thia-[9.9](2,6,3,5)-pyrazino­phane (**L1**), with copper(II) dibromide led to the formation of a binuclear complex, [(μ_2_-**L1**)di­bromodo dicopper(II)] dibromide, (**I**); see Fig. 2 ▸. The complex possesses inversion symmetry with the pyrazine ring being situated about a center of symmetry. Selected bond distances and angles are given in Table 1 ▸. The ligand coordinates to the copper(II) atoms in a bis-tetra­dentate manner. The symmetry related Cu atoms have a fivefold NS_3_Br coordination environment with a distorted shape, as indicated by the fivefold index parameter τ_5_ of 0.38 (τ_5_ = 0 for an ideal square-pyramidal coordination sphere, and = 1 for an ideal trigonal–pyramidal coordination sphere; Addison *et al.*, 1984 ▸). There are four five-membered chelate rings; Cu1/N1/C2/C3/S1 and Cu1/N1/C1/C8/S3 which are inclined by *ca* 90° to chelate rings Cu1/S1/C4/C5/S2 and Cu1/S2/C6/C7/S3 (Fig. 2 ▸).

**Figure 3:**
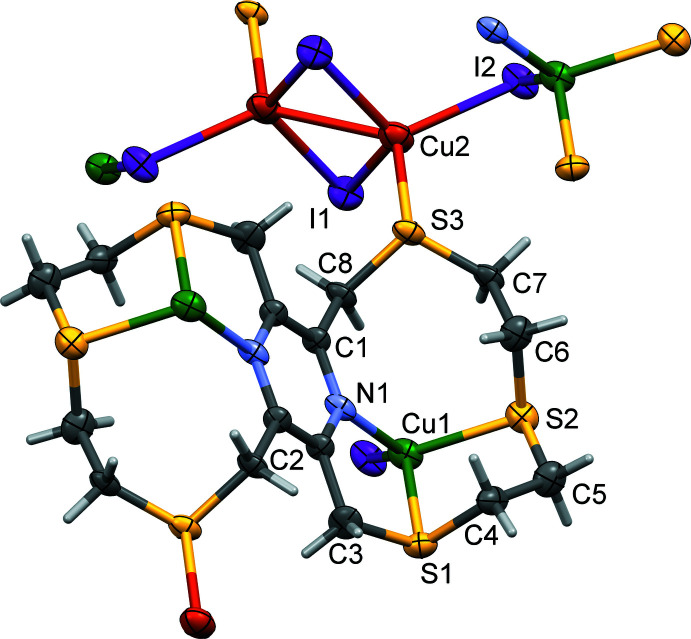
A view of the mol­ecular structure of complex **II**, with atom labelling for the asymmetric unit; symmetry codes: (i) *x* + 1, *y*, z; (ii) −*x* + 1, −*y* + 1, −*z*; (iii) *x* − 1, *y*, *z*. Displacement ellipsoids are drawn at the 50% probability level. (Atom Cu1 is green, while atom Cu2 is orange.)

Reaction of **L1** with copper(I) iodide also gave a binuclear complex, which is bridged by a Cu_2_I_2_ unit to form a two-dimensional coordination polymer, poly-[(μ_2_-**L1**)di­iodido­dicopper(I)di(μ-iodido)­dicopper(I)], (**II**); see Fig. 3 ▸. The binuclear complex possesses inversion symmetry with the pyrazine ring being located about a center of symmetry. The Cu_2_I_2_ unit is also located about an inversion center. Selected bond distances and angles are given in Table 2 ▸. The two independent copper(I) atoms, Cu1 and Cu2, are both fourfold coordinate. Atom Cu1 coordinates to the ligand **L1** in a tridentate fashion and has an NS_2_I coordination environment. The fourfold index parameter τ_4_ is 0.77 indicating a very irregular shape (τ_4_ = 1 for a perfect tetra­hedral environment, 0 for a perfect square-planar environment and 0.85 for a perfect trigonal–pyramidal environment; Yang *et al.*, 2007 ▸). There are three chelate rings, two of which are five-membered (Cu1/N1/C2/C3/S1 and Cu1/S1/C4/C5/S2) and one eight-membered (Cu1/N1/C1/C8/S3/C7/C6/S2). The second copper(I) atom, Cu2, coordinates to **L1** in a monodentate fashion and has an SI_3_ environment with an almost perfect tetra­hedral geometry; here the fourfold index parameter τ_4_ is 0.91.

The Cu1—N1 bond lengths in the two complexes, 2.046 (6) Å in **I** and 2.095 (10) Å in **II**, are significantly different (Linden, 2020 ▸). They have a difference of 0.049 (12) Å so differ by 4.1σ (*i.e*., 0.049 Å = 0.012 Å × 4.1). In **I**, the bond length Cu1—S2 of 2.455 (2) Å is significantly longer than bond lengths Cu1—S1 [2.346 (2) Å] and Cu1—S3 [2.333 (2) Å]. In **II**, bond lengths Cu1—S1 and Cu1—S2, involving the five-membered chelate rings, *viz*. 2.342 (4) and 2.331 (4) Å, respectively, are similar to those in **I**, while bond length Cu2—S3 [2.359 (4) Å] is only slightly longer. The bridging Cu2—Cu2^i^ distance in the Cu_2_I_2_ unit in **II** is 2.663 (4) Å (Table 2 ▸), considerably shorter than the same distance observed in complex **CuI-L2** [2.776 (1) Å] [Fig. 1 ▸ *a*; Assoumatine & Stoeckli-Evans, 2020*b* ▸].

## Supra­molecular features   

**Figure 4:**
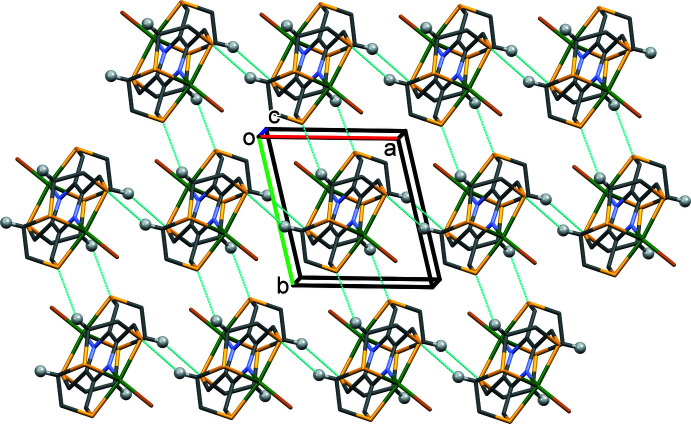
A view along the *c* axis of the crystal packing of **I**. The C—H⋯S hydrogen bonds are shown as dashed lines (see Table 3 ▸). For clarity, the Br^−^ anion and the H atoms not involved in these inter­molecular inter­actions have been omitted.

**Figure 5:**
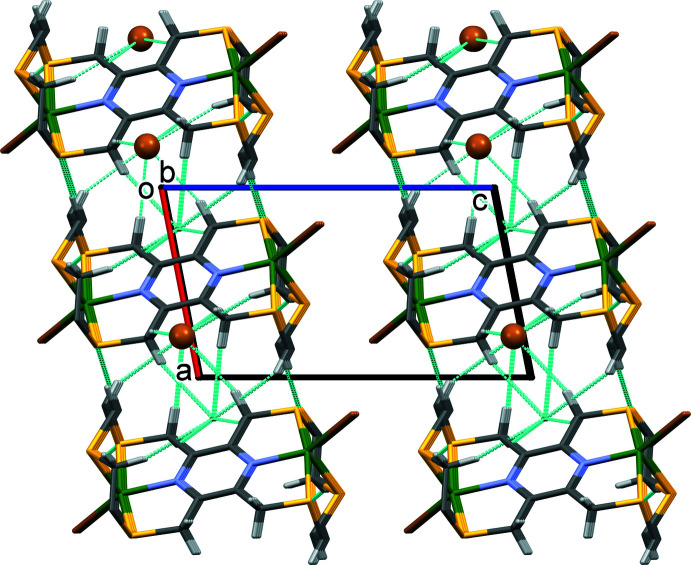
A view along the *b* axis of the crystal packing of **I**. The C—H⋯S and C—H⋯Br^−^ hydrogen bonds (Table 3 ▸) are shown as dashed lines (see Table 3 ▸). For clarity, only the H atoms involved in these inter­molecular inter­actions have been included.

In the crystal of **I**, the cations are linked by pairs of C6—H6*B*⋯S1^i^ hydrogen bonds to form chains along the *a-*axis direction. Chains are also formed along the *b*-axis direction *via* C5—H5*A*⋯S3^ii^ hydrogen bonds (Table 3 ▸). These inter­actions result in the formation of a supra­molecular network that lies parallel to the *ab* plane (Fig. 4 ▸). There are also a large number of C—H⋯Br contacts present involving the anion, Br2^−^, strengthening the supra­molecular network (Fig. 5 ▸ and Table 3 ▸). There are no significant inter-layer contacts present in the crystal.

**Figure 6:**
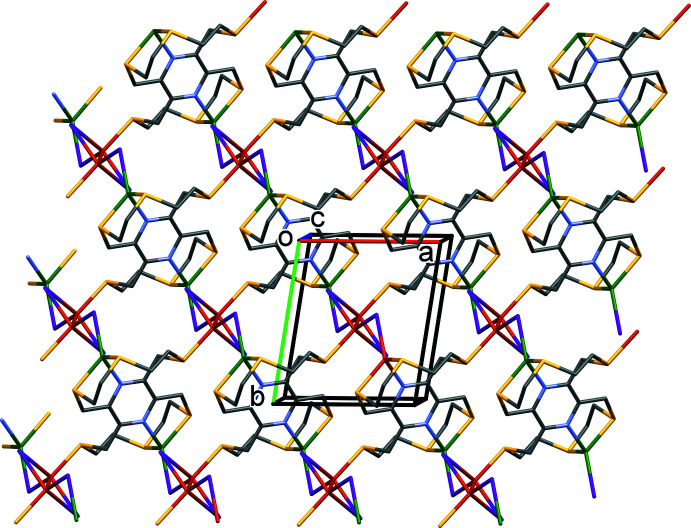
A view along the *c* axis of the two-dimensional structure of complex **II**. For clarity, H atoms have been omitted.

**Figure 7:**
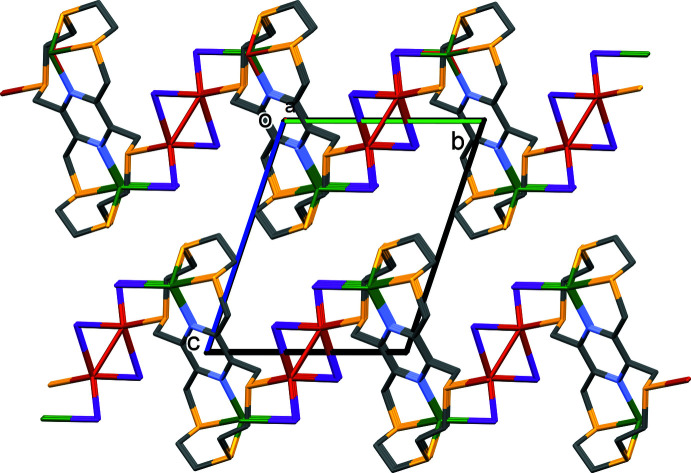
A view along the *a* axis of the crystal packing of complex **II**. For clarity, H atoms have been omitted.

In the crystal of **II**, the two-dimensional coordination polymers lie parallel to the (001) plane, as shown in Fig. 6 ▸. There are no significant inter-layer contacts present in the crystal (Fig. 7 ▸).

## Database survey   

A search of the Cambridge Structural Database (CSD, Version 5.41, last update March 2020; Groom *et al.*, 2016 ▸) for tri- or hexa-thia­benzeno­phane ligands gave only three hits. They include the tri­thia­benzeno­phane ligand, 2,5,8-tri­thia­(9)-*m*-benzeno­phane (CSD refcode VEYNES; Groot & Loeb, 1990 ▸), and a palladium and a silver complex of the same ligand, *viz*. di­chloro­[2,5,8-tri­thia­(9)-*m*-benzeno­phane]palladium(II) (KOMNOP; Groot *et al.*, 1991 ▸), a mononuclear complex, and poly[[2,5,8-tri­thia­(9)-*m*-cyclo­phane-*S,S′,S′′*]silver(I) tri­fluoro­methyl­sulfonate aceto­nitrile solvate] (ZIDPEH; Casabo *et al.*, 1995 ▸), a two-dimensional coordination polymer. In KOMNOP, the ligand coordinates in a bidentate manner. The palladium(II) atom is fourfold S_2_Cl_2_ coordinate with a square-planar environment (index parameter τ_4_ is 0.04), In ZIDPEH, the ligand coordinates in a bridging μ_3_-monodentate manner. The silver(I) atom is fivefold NOS_3_ coordinate with an irregular shape (index parameter τ_5_ is 0.56).

A search for benzeno­phane ligands similar to **L2** and **L3** gave zero hits for **L2** and ten hits for **L3**. The latter compounds have been compared in a recent article (Assoumatine & Stoeckli-Evans, 2020*d* ▸), which also describes the syntheses and crystal structures of both **L2** and **L3**.

## Synthesis and crystallization   

The synthesis and crystal structure of the ligand 2,5,8,11,14,17-hexa­thia-[9.9](2,6,3,5)-pyrazino­phane (**L1**), have been reported (Assoumatine & Stoeckli-Evans, 2020*a* ▸).

**Synthesis of complex [(μ_2_-L1)di­bromodo dicopper(II)] dibromide (I):** A solution of **L1** (15 mg, 0.03 mmol) in CHCl_3_ (10 ml) was introduced into a 16 mm diameter glass tube and layered with MeCN (2 ml) as a buffer zone. Then a solution of CuBr_2_ (7 mg, 0.03 mmol) in MeCN (5 ml) was added gently to avoid possible mixing. The glass tube was sealed and left in the dark at room temperature for at least 3 weeks, whereupon brown crystals of complex **I** were isolated in the buffer zone.

**Synthesis of complex poly-[(μ_2_-L1)di­iodido-dicopper(I)-di(μ-iodido)-dicopper(I)] (II):** A solution of **L1** (15 mg, 0.03 mmol) in CH_2_Cl_2_ (5 ml) was introduced into a 16 mm diameter glass tube and layered with MeCN (2 ml) as a buffer zone. A solution of CuI (6 mg, 0.03 mmol) in MeCN (5 ml) was added gently to avoid possible mixing. The glass tube was sealed under an atmosphere of nitro­gen and left in the dark at room temperature for at least 3 weeks, whereupon small orange crystals of complex **II** were isolated in the buffer zone.

## Refinement   

Crystal data, data collection and structure refinement details are summarized in Table 4 ▸. The C-bound H atoms were included in calculated positions and treated as riding on their parent atoms: C—H = 0.98 Å for **I** and 0.97 Å for **II**, with *U*_iso_(H) = 1.2*U*_eq_(C).

Intensity data were measured using a STOE IPDS-1 one-circle diffractometer. For the triclinic system often only 93% of the Ewald sphere is accessible, which explains why the alerts diffrn_reflns_laue_measured_fraction_full value (0.94) below minimum (0.95) for both compounds **I** and **II** are given. This involves 145 random reflections out of the expected 2336 for the IUCr cutoff limit of sin θ/λ = 0.60 for **I**, and 155 random reflections out of the expected 2600 reflections for **II**. The residual electron-density peaks are approximately 1Å from the halogen atoms in both structures.

## Supplementary Material

Crystal structure: contains datablock(s) I, II, Global. DOI: 10.1107/S2056989020007161/pk2634sup1.cif

Structure factors: contains datablock(s) I. DOI: 10.1107/S2056989020007161/pk2634Isup2.hkl

Structure factors: contains datablock(s) II. DOI: 10.1107/S2056989020007161/pk2634IIsup3.hkl

CCDC references: 2006571, 2006572

Additional supporting information:  crystallographic information; 3D view; checkCIF report

## Figures and Tables

**Table 1:** Selected geometric parameters (Å, °) for **I** [Chem scheme1]

Cu1—N1	2.046 (6)	Cu1—S3	2.333 (2)
Cu1—S1	2.346 (2)	Cu1—Br1	2.3672 (11)
Cu1—S2	2.4549 (18)		
			
N1—Cu1—S1	85.26 (18)	S3—Cu1—Br1	94.97 (6)
N1—Cu1—S3	85.38 (18)	N1—Cu1—S2	104.48 (15)
S3—Cu1—S1	168.54 (7)	S1—Cu1—S2	88.79 (7)
N1—Cu1—Br1	145.45 (15)	S3—Cu1—S2	87.17 (7)
S1—Cu1—Br1	96.48 (6)	Br1—Cu1—S2	110.05 (6)

**Table 2:** Selected geometric parameters (Å, °) for **II** [Chem scheme1]

Cu1—N1	2.095 (10)	Cu2—I1	2.665 (2)
Cu1—S1	2.342 (4)	Cu2—I2	2.6166 (19)
Cu1—S2	2.331 (4)	I1—Cu2^ii^	2.675 (2)
Cu1—I2^i^	2.5193 (18)	Cu2—Cu2^ii^	2.663 (4)
Cu2—S3	2.359 (4)		
			
N1—Cu1—S2	110.2 (3)	S3—Cu2—I1^ii^	99.22 (11)
N1—Cu1—S1	85.3 (3)	I2—Cu2—I1^ii^	112.01 (7)
S2—Cu1—S1	91.74 (14)	I1—Cu2—I1^ii^	120.18 (7)
N1—Cu1—I2^i^	121.1 (3)	Cu2—I1—Cu2^ii^	59.82 (7)
S2—Cu1—I2^i^	112.48 (12)	Cu1^iii^—I2—Cu2	94.93 (7)
S1—Cu1—I2^i^	130.61 (10)	Cu2^ii^—Cu2—I1	60.27 (7)
S3—Cu2—I2	109.54 (11)	Cu2^ii^—Cu2—I1^ii^	59.91 (7)
S3—Cu2—I1	105.86 (10)	S3—Cu2—Cu2^ii^	115.75 (13)
I2—Cu2—I1	109.04 (8)	I2—Cu2—Cu2^ii^	134.68 (11)

Symmetry codes: (i) 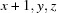; (ii) 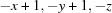; (iii) 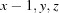.

**Table 3:** Hydrogen-bond geometry (Å, °) for **I** [Chem scheme1]

*D*—H⋯*A*	*D*—H	H⋯*A*	*D*⋯*A*	*D*—H⋯*A*
C6—H6*B*⋯S1^i^	0.98	2.85	3.753 (9)	154
C5—H5*A*⋯S3^ii^	0.98	2.81	3.634 (8)	143
C3—H3*A*⋯Br2^iii^	0.98	2.86	3.814 (8)	165
C3—H3*B*⋯Br2^ii^	0.98	2.83	3.770 (7)	160
C5—H5*B*⋯Br2^iv^	0.98	2.87	3.821 (7)	164
C7—H7*B*⋯Br2^i^	0.98	2.82	3.646 (8)	142
C8—H8*A*⋯Br2^i^	0.98	2.84	3.769 (9)	159
C8—H8*B*⋯Br2^v^	0.98	2.89	3.713 (7)	142

Symmetry codes: (i) 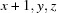; (ii) 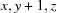; (iii) 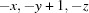; (iv) 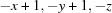; (v) 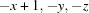.

**Table 4:** Experimental details

	**I**	**II**
Crystal data		
Chemical formula	[Cu_2_Br_2_(C_16_H_24_N_2_S_6_)]Br_2_	[Cu_4_I_4_(C_16_H_24_N_2_S_6_)]
*M*_r_	883.45	1198.49
Crystal system, space group	Triclinic, *P* 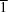	Triclinic, *P* 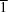
Temperature (K)	223	293
*a*, *b*, *c* (Å)	7.2090 (7), 8.1422 (8), 12.3904 (14)	7.7713 (8), 8.9456 (9), 11.2464 (14)
α, β, γ (°)	71.842 (12), 74.702 (12), 72.694 (12)	106.839 (13), 104.644 (13), 93.412 (12)
*V* (Å^3^)	647.93 (13)	716.53 (15)
*Z*	1	1
Radiation type	Mo *K*α	Mo *K*α
μ (mm^−1^)	8.30	7.69
Crystal size (mm)	0.20 × 0.20 × 0.03	0.30 × 0.20 × 0.05

Data collection		
Diffractometer	Stoe IPDS 1	Stoe IPDS 1
Absorption correction	Multi-scan (*MULABS*; Spek, 2020 ▸)	Multi-scan (*MULABS*; Spek, 2020 ▸)
*T*_min_, *T*_max_	0.421, 1.000	0.435, 1.000
No. of measured, independent and observed [*I* > 2σ(*I*)] reflections	5057, 2320, 1843	5260, 2505, 1698
*R*_int_	0.076	0.100
(sin θ/λ)_max_ (Å^−1^)	0.613	0.606

Refinement		
*R*[*F* ^2^ > 2σ(*F* ^2^)], *wR*(*F* ^2^), *S*	0.060, 0.153, 1.01	0.070, 0.183, 0.95
No. of reflections	2320	2505
No. of parameters	106	127
H-atom treatment	H-atom parameters constrained	H-atom parameters constrained
Δρ_max_, Δρ_min_ (e Å^−3^)	1.65, −1.67	2.25, −2.58

Computer programs: *EXPOSE*, *CELL* and *INTEGRATE* in *IPDS-I* (Stoe & Cie, 1997 ▸), *SHELXS97* (Sheldrick, 2008 ▸), *Mercury* (Macrae *et al.*, 2020 ▸), *SHELXL2018/3* (Sheldrick, 2015 ▸), *PLATON* (Spek, 2020 ▸) and *publCIF* (Westrip, 2010 ▸).

## supplementary crystallographic information

### [µ2-2,5,8,11,14,17-Hexathia-[9.9](2,6,3,5)-pyrazinophane]bis[bromidocopper(II)] dibromide (I) Crystal data

**Table:**

[Cu_2_Br_2_(C_16_H_24_N_2_S_6_)]Br_2_	*Z* = 1
*M**_r_* = 883.45	*F*(000) = 428
Triclinic, *P*1	*D*_x_ = 2.264 Mg m^−^^3^
*a* = 7.2090 (7) Å	Mo *K*α radiation, λ = 0.71073 Å
*b* = 8.1422 (8) Å	Cell parameters from 5000 reflections
*c* = 12.3904 (14) Å	θ = 2.7–25.8°
α = 71.842 (12)°	µ = 8.30 mm^−^^1^
β = 74.702 (12)°	*T* = 223 K
γ = 72.694 (12)°	Plate, brown
*V* = 647.93 (13) Å^3^	0.20 × 0.20 × 0.03 mm

### [µ2-2,5,8,11,14,17-Hexathia-[9.9](2,6,3,5)-pyrazinophane]bis[bromidocopper(II)] dibromide (I) Data collection

**Table:**

STOE IPDS 1 diffractometer	2320 independent reflections
Radiation source: fine-focus sealed tube	1843 reflections with *I* > 2σ(*I*)
Plane graphite monochromator	*R*_int_ = 0.076
φ rotation scans	θ_max_ = 25.8°, θ_min_ = 2.7°
Absorption correction: multi-scan (MULABS; Spek, 2020)	*h* = −8→8
*T*_min_ = 0.421, *T*_max_ = 1.000	*k* = −9→9
5057 measured reflections	*l* = −15→15

### [µ2-2,5,8,11,14,17-Hexathia-[9.9](2,6,3,5)-pyrazinophane]bis[bromidocopper(II)] dibromide (I) Refinement

**Table:**

Refinement on *F*^2^	Primary atom site location: structure-invariant direct methods
Least-squares matrix: full	Secondary atom site location: difference Fourier map
*R*[*F*^2^ > 2σ(*F*^2^)] = 0.060	Hydrogen site location: inferred from neighbouring sites
*wR*(*F*^2^) = 0.153	H-atom parameters constrained
*S* = 1.01	*w* = 1/[σ^2^(*F*_o_^2^) + (0.1019*P*)^2^] where *P* = (*F*_o_^2^ + 2*F*_c_^2^)/3
2320 reflections	(Δ/σ)_max_ < 0.001
106 parameters	Δρ_max_ = 1.65 e Å^−^^3^
0 restraints	Δρ_min_ = −1.67 e Å^−^^3^

### [µ2-2,5,8,11,14,17-Hexathia-[9.9](2,6,3,5)-pyrazinophane]bis[bromidocopper(II)] dibromide (I) Special details

**Table:**

Geometry. All esds (except the esd in the dihedral angle between two l.s. planes) are estimated using the full covariance matrix. The cell esds are taken into account individually in the estimation of esds in distances, angles and torsion angles; correlations between esds in cell parameters are only used when they are defined by crystal symmetry. An approximate (isotropic) treatment of cell esds is used for estimating esds involving l.s. planes.

### [µ2-2,5,8,11,14,17-Hexathia-[9.9](2,6,3,5)-pyrazinophane]bis[bromidocopper(II)] dibromide (I) Fractional atomic coordinates and isotropic or equivalent isotropic displacement parameters (Å^2^)

**Table:**

	*x*	*y*	*z*	*U*_iso_*/*U*_eq_	
Br1	0.19307 (12)	0.19827 (12)	0.45400 (7)	0.0328 (3)	
Cu1	0.38840 (13)	0.35871 (11)	0.29477 (7)	0.0163 (3)	
S1	0.1670 (3)	0.6379 (2)	0.28528 (15)	0.0215 (4)	
S2	0.6115 (3)	0.4639 (2)	0.36310 (15)	0.0197 (4)	
S3	0.6481 (3)	0.1120 (2)	0.27313 (15)	0.0175 (4)	
N1	0.4546 (9)	0.4439 (7)	0.1184 (5)	0.0156 (7)	
C1	0.5922 (11)	0.3381 (9)	0.0573 (6)	0.0156 (7)	
C2	0.3618 (10)	0.6046 (9)	0.0628 (6)	0.0156 (7)	
C3	0.2054 (11)	0.7229 (9)	0.1295 (6)	0.0202 (16)	
H3A	0.080095	0.744824	0.104611	0.024*	
H3B	0.240502	0.837592	0.109580	0.024*	
C4	0.3042 (11)	0.7561 (10)	0.3270 (6)	0.0213 (9)	
H4A	0.262709	0.883424	0.290981	0.026*	
H4B	0.267942	0.739951	0.411020	0.026*	
C5	0.5303 (11)	0.6978 (9)	0.2940 (6)	0.0156 (7)	
H5A	0.592228	0.768345	0.318918	0.019*	
H5B	0.570091	0.717761	0.209820	0.019*	
C6	0.8280 (12)	0.3836 (9)	0.2624 (6)	0.0213 (9)	
H6A	0.808898	0.438922	0.182395	0.026*	
H6B	0.945536	0.410333	0.272072	0.026*	
C7	0.8506 (11)	0.1809 (9)	0.2918 (7)	0.0223 (16)	
H7A	0.867485	0.130400	0.372431	0.027*	
H7B	0.971846	0.130109	0.243159	0.027*	
C8	0.6904 (12)	0.1574 (9)	0.1173 (6)	0.0213 (9)	
H8A	0.833237	0.139237	0.087755	0.026*	
H8B	0.644904	0.070380	0.097392	0.026*	
Br2	0.21653 (11)	0.20772 (10)	0.02398 (7)	0.0267 (3)	

### [µ2-2,5,8,11,14,17-Hexathia-[9.9](2,6,3,5)-pyrazinophane]bis[bromidocopper(II)] dibromide (I) Atomic displacement parameters (Å^2^)

**Table:**

	*U*^11^	*U*^22^	*U*^33^	*U*^12^	*U*^13^	*U*^23^
Br1	0.0270 (5)	0.0335 (5)	0.0267 (5)	−0.0116 (4)	−0.0043 (3)	0.0114 (3)
Cu1	0.0194 (5)	0.0085 (5)	0.0194 (5)	−0.0036 (4)	−0.0032 (4)	−0.0013 (3)
S1	0.0215 (10)	0.0172 (10)	0.0201 (9)	0.0018 (8)	−0.0013 (7)	−0.0053 (7)
S2	0.0281 (10)	0.0115 (9)	0.0223 (9)	−0.0028 (8)	−0.0095 (8)	−0.0060 (7)
S3	0.0270 (10)	0.0051 (8)	0.0195 (8)	−0.0025 (8)	−0.0085 (7)	0.0005 (6)
N1	0.0223 (18)	0.0065 (15)	0.0219 (16)	−0.0034 (14)	−0.0088 (13)	−0.0053 (12)
C1	0.0223 (18)	0.0065 (15)	0.0219 (16)	−0.0034 (14)	−0.0088 (13)	−0.0053 (12)
C2	0.0223 (18)	0.0065 (15)	0.0219 (16)	−0.0034 (14)	−0.0088 (13)	−0.0053 (12)
C3	0.029 (4)	0.012 (4)	0.014 (3)	0.008 (3)	−0.007 (3)	−0.006 (3)
C4	0.031 (3)	0.011 (2)	0.022 (2)	−0.002 (2)	−0.0101 (19)	−0.0047 (17)
C5	0.0223 (18)	0.0065 (15)	0.0219 (16)	−0.0034 (14)	−0.0088 (13)	−0.0053 (12)
C6	0.031 (3)	0.011 (2)	0.022 (2)	−0.002 (2)	−0.0101 (19)	−0.0047 (17)
C7	0.024 (4)	0.011 (4)	0.035 (4)	0.002 (3)	−0.020 (3)	−0.004 (3)
C8	0.031 (3)	0.011 (2)	0.022 (2)	−0.002 (2)	−0.0101 (19)	−0.0047 (17)
Br2	0.0247 (4)	0.0210 (4)	0.0385 (5)	−0.0073 (4)	−0.0041 (3)	−0.0127 (3)

### [µ2-2,5,8,11,14,17-Hexathia-[9.9](2,6,3,5)-pyrazinophane]bis[bromidocopper(II)] dibromide (I) Geometric parameters (Å, º)

**Table:**

Cu1—N1	2.046 (6)	C2—C3	1.497 (9)
Cu1—S1	2.346 (2)	C3—H3A	0.9800
Cu1—S2	2.4549 (18)	C3—H3B	0.9800
Cu1—S3	2.333 (2)	C4—C5	1.536 (10)
Cu1—Br1	2.3672 (11)	C4—H4A	0.9800
S1—C3	1.811 (7)	C4—H4B	0.9800
S1—C4	1.822 (7)	C5—H5A	0.9800
S2—C6	1.815 (8)	C5—H5B	0.9800
S2—C5	1.814 (7)	C6—C7	1.542 (9)
S3—C7	1.802 (7)	C6—H6A	0.9800
S3—C8	1.808 (7)	C6—H6B	0.9800
N1—C1	1.342 (9)	C7—H7A	0.9800
N1—C2	1.340 (9)	C7—H7B	0.9800
C1—C2^i^	1.394 (10)	C8—H8A	0.9800
C1—C8	1.482 (11)	C8—H8B	0.9800
			
N1—Cu1—S1	85.26 (18)	S1—C3—H3B	108.4
N1—Cu1—S3	85.38 (18)	H3A—C3—H3B	107.5
S3—Cu1—S1	168.54 (7)	C5—C4—S1	115.1 (5)
N1—Cu1—Br1	145.45 (15)	C5—C4—H4A	108.5
S1—Cu1—Br1	96.48 (6)	S1—C4—H4A	108.5
S3—Cu1—Br1	94.97 (6)	C5—C4—H4B	108.5
N1—Cu1—S2	104.48 (15)	S1—C4—H4B	108.5
S1—Cu1—S2	88.79 (7)	H4A—C4—H4B	107.5
S3—Cu1—S2	87.17 (7)	C4—C5—S2	109.4 (4)
Br1—Cu1—S2	110.05 (6)	C4—C5—H5A	109.8
C3—S1—C4	102.4 (3)	S2—C5—H5A	109.8
C3—S1—Cu1	98.5 (2)	C4—C5—H5B	109.8
C4—S1—Cu1	101.4 (3)	S2—C5—H5B	109.8
C6—S2—C5	104.4 (3)	H5A—C5—H5B	108.3
C6—S2—Cu1	93.8 (2)	C7—C6—S2	105.4 (5)
C5—S2—Cu1	96.5 (2)	C7—C6—H6A	110.7
C7—S3—C8	100.9 (4)	S2—C6—H6A	110.7
C7—S3—Cu1	101.0 (2)	C7—C6—H6B	110.7
C8—S3—Cu1	97.8 (3)	S2—C6—H6B	110.7
C1—N1—C2	119.4 (6)	H6A—C6—H6B	108.8
C1—N1—Cu1	119.9 (5)	C6—C7—S3	115.5 (5)
C2—N1—Cu1	120.7 (5)	C6—C7—H7A	108.4
N1—C1—C2^i^	120.2 (7)	S3—C7—H7A	108.4
N1—C1—C8	119.9 (6)	C6—C7—H7B	108.4
C2^i^—C1—C8	119.9 (6)	S3—C7—H7B	108.4
N1—C2—C1^i^	120.4 (6)	H7A—C7—H7B	107.5
N1—C2—C3	120.1 (6)	C1—C8—S3	115.4 (5)
C1^i^—C2—C3	119.5 (7)	C1—C8—H8A	108.4
C2—C3—S1	115.3 (5)	S3—C8—H8A	108.4
C2—C3—H3A	108.4	C1—C8—H8B	108.4
S1—C3—H3A	108.4	S3—C8—H8B	108.4
C2—C3—H3B	108.4	H8A—C8—H8B	107.5
			
C2—N1—C1—C2^i^	0.2 (10)	Cu1—S1—C4—C5	30.4 (6)
Cu1—N1—C1—C2^i^	−179.9 (4)	S1—C4—C5—S2	−59.9 (6)
C2—N1—C1—C8	−177.6 (5)	C6—S2—C5—C4	148.0 (5)
Cu1—N1—C1—C8	2.3 (8)	Cu1—S2—C5—C4	52.4 (4)
C1—N1—C2—C1^i^	−0.2 (10)	C5—S2—C6—C7	−158.1 (5)
Cu1—N1—C2—C1^i^	179.9 (4)	Cu1—S2—C6—C7	−60.3 (5)
C1—N1—C2—C3	178.9 (6)	S2—C6—C7—S3	62.3 (6)
Cu1—N1—C2—C3	−1.0 (8)	C8—S3—C7—C6	74.9 (6)
N1—C2—C3—S1	3.6 (8)	Cu1—S3—C7—C6	−25.4 (6)
C1^i^—C2—C3—S1	−177.3 (5)	N1—C1—C8—S3	−11.6 (8)
C4—S1—C3—C2	99.9 (5)	C2^i^—C1—C8—S3	170.6 (5)
Cu1—S1—C3—C2	−3.8 (5)	C7—S3—C8—C1	−89.9 (6)
C3—S1—C4—C5	−71.0 (6)	Cu1—S3—C8—C1	13.0 (5)

Symmetry code: (i) −*x*+1, −*y*+1, −*z*.

### [µ2-2,5,8,11,14,17-Hexathia-[9.9](2,6,3,5)-pyrazinophane]bis[bromidocopper(II)] dibromide (I) Hydrogen-bond geometry (Å, º)

**Table:**

*D*—H···*A*	*D*—H	H···*A*	*D*···*A*	*D*—H···*A*
C6—H6*B*···S1^ii^	0.98	2.85	3.753 (9)	154
C5—H5*A*···S3^iii^	0.98	2.81	3.634 (8)	143
C3—H3*A*···Br2^iv^	0.98	2.86	3.814 (8)	165
C3—H3*B*···Br2^iii^	0.98	2.83	3.770 (7)	160
C5—H5*B*···Br2^i^	0.98	2.87	3.821 (7)	164
C7—H7*B*···Br2^ii^	0.98	2.82	3.646 (8)	142
C8—H8*A*···Br2^ii^	0.98	2.84	3.769 (9)	159
C8—H8*B*···Br2^v^	0.98	2.89	3.713 (7)	142

Symmetry codes: (i) −*x*+1, −*y*+1, −*z*; (ii) *x*+1, *y*, *z*; (iii) *x*, *y*+1, *z*; (iv) −*x*, −*y*+1, −*z*; (v) −*x*+1, −*y*, −*z*.

### Poly[[µ2-2,5,8,11,14,17-hexathia-[9.9](2,6,3,5)-pyrazinophane]tetra-µ-iodido-tetracopper(I)] (II) Crystal data

**Table:**

[Cu_4_I_4_(C_16_H_24_N_2_S_6_)]	*Z* = 1
*M**_r_* = 1198.49	*F*(000) = 558
Triclinic, *P*1	*D*_x_ = 2.777 Mg m^−^^3^
*a* = 7.7713 (8) Å	Mo *K*α radiation, λ = 0.71073 Å
*b* = 8.9456 (9) Å	Cell parameters from 4652 reflections
*c* = 11.2464 (14) Å	θ = 2.0–25.8°
α = 106.839 (13)°	µ = 7.69 mm^−^^1^
β = 104.644 (13)°	*T* = 293 K
γ = 93.412 (12)°	Plate, orange
*V* = 716.53 (15) Å^3^	0.30 × 0.20 × 0.05 mm

### Poly[[µ2-2,5,8,11,14,17-hexathia-[9.9](2,6,3,5)-pyrazinophane]tetra-µ-iodido-tetracopper(I)] (II) Data collection

**Table:**

STOE IPDS 1 diffractometer	2505 independent reflections
Radiation source: fine-focus sealed tube	1698 reflections with *I* > 2σ(*I*)
Plane graphite monochromator	*R*_int_ = 0.100
φ rotation scans	θ_max_ = 25.5°, θ_min_ = 2.4°
Absorption correction: multi-scan (MULABS; Spek, 2020)	*h* = −9→9
*T*_min_ = 0.435, *T*_max_ = 1.000	*k* = −10→10
5260 measured reflections	*l* = −13→13

### Poly[[µ2-2,5,8,11,14,17-hexathia-[9.9](2,6,3,5)-pyrazinophane]tetra-µ-iodido-tetracopper(I)] (II) Refinement

**Table:**

Refinement on *F*^2^	Primary atom site location: structure-invariant direct methods
Least-squares matrix: full	Secondary atom site location: difference Fourier map
*R*[*F*^2^ > 2σ(*F*^2^)] = 0.070	Hydrogen site location: inferred from neighbouring sites
*wR*(*F*^2^) = 0.183	H-atom parameters constrained
*S* = 0.95	*w* = 1/[σ^2^(*F*_o_^2^) + (0.110*P*)^2^] where *P* = (*F*_o_^2^ + 2*F*_c_^2^)/3
2505 reflections	(Δ/σ)_max_ < 0.001
127 parameters	Δρ_max_ = 2.25 e Å^−^^3^
0 restraints	Δρ_min_ = −2.58 e Å^−^^3^

### Poly[[µ2-2,5,8,11,14,17-hexathia-[9.9](2,6,3,5)-pyrazinophane]tetra-µ-iodido-tetracopper(I)] (II) Special details

**Table:**

Geometry. All esds (except the esd in the dihedral angle between two l.s. planes) are estimated using the full covariance matrix. The cell esds are taken into account individually in the estimation of esds in distances, angles and torsion angles; correlations between esds in cell parameters are only used when they are defined by crystal symmetry. An approximate (isotropic) treatment of cell esds is used for estimating esds involving l.s. planes.

### Poly[[µ2-2,5,8,11,14,17-hexathia-[9.9](2,6,3,5)-pyrazinophane]tetra-µ-iodido-tetracopper(I)] (II) Fractional atomic coordinates and isotropic or equivalent isotropic displacement parameters (Å^2^)

**Table:**

	*x*	*y*	*z*	*U*_iso_*/*U*_eq_	
I1	0.73534 (12)	0.70224 (11)	0.11663 (9)	0.0396 (3)	
I2	0.34990 (12)	0.56223 (11)	0.29340 (9)	0.0370 (3)	
Cu1	1.2404 (2)	0.2890 (2)	0.28830 (16)	0.0332 (4)	
Cu2	0.5027 (2)	0.4653 (2)	0.10778 (18)	0.0415 (5)	
S1	1.3999 (4)	0.0851 (4)	0.3255 (3)	0.0326 (8)	
S2	1.1079 (5)	0.2923 (4)	0.4531 (3)	0.0349 (8)	
S3	0.6749 (4)	0.2663 (4)	0.1450 (3)	0.0296 (7)	
N1	1.0914 (12)	0.1189 (12)	0.1149 (10)	0.025 (2)	
C1	0.9371 (16)	0.1373 (15)	0.0368 (12)	0.0238 (18)	
C2	1.1538 (15)	−0.0213 (15)	0.0762 (11)	0.0238 (18)	
C3	1.3271 (19)	−0.0448 (18)	0.1606 (13)	0.038 (3)	
H3A	1.421167	−0.033142	0.120089	0.046*	
H3B	1.314897	−0.152610	0.162526	0.046*	
C4	1.2385 (17)	0.0077 (18)	0.3947 (13)	0.035 (3)	
H4A	1.126049	−0.037376	0.327911	0.042*	
H4B	1.284935	−0.075140	0.427765	0.042*	
C5	1.206 (2)	0.1374 (18)	0.5023 (14)	0.039 (2)	
H5A	1.319763	0.181893	0.567821	0.047*	
H5B	1.127970	0.092387	0.541897	0.047*	
C6	0.8751 (19)	0.2106 (18)	0.3709 (14)	0.039 (2)	
H6A	0.868179	0.113955	0.301594	0.047*	
H6B	0.817806	0.185297	0.431304	0.047*	
C7	0.7789 (16)	0.3263 (16)	0.3162 (12)	0.030 (2)	
H7A	0.864140	0.420800	0.338968	0.036*	
H7B	0.686666	0.355435	0.359299	0.036*	
C8	0.8623 (16)	0.2892 (16)	0.0814 (13)	0.030 (2)	
H8A	0.824277	0.329504	0.008985	0.036*	
H8B	0.956964	0.366690	0.147795	0.036*	

### Poly[[µ2-2,5,8,11,14,17-hexathia-[9.9](2,6,3,5)-pyrazinophane]tetra-µ-iodido-tetracopper(I)] (II) Atomic displacement parameters (Å^2^)

**Table:**

	*U*^11^	*U*^22^	*U*^33^	*U*^12^	*U*^13^	*U*^23^
I1	0.0407 (5)	0.0292 (6)	0.0494 (6)	0.0003 (4)	0.0136 (4)	0.0134 (4)
I2	0.0437 (5)	0.0156 (5)	0.0585 (6)	0.0031 (4)	0.0271 (4)	0.0110 (4)
Cu1	0.0348 (8)	0.0176 (10)	0.0487 (10)	0.0019 (7)	0.0144 (7)	0.0110 (7)
Cu2	0.0424 (10)	0.0387 (12)	0.0546 (12)	0.0177 (8)	0.0220 (8)	0.0219 (9)
S1	0.0277 (15)	0.030 (2)	0.0386 (18)	0.0065 (13)	0.0051 (13)	0.0109 (15)
S2	0.0374 (17)	0.025 (2)	0.042 (2)	0.0028 (14)	0.0146 (14)	0.0084 (15)
S3	0.0284 (15)	0.0216 (19)	0.045 (2)	0.0109 (13)	0.0161 (14)	0.0138 (14)
N1	0.025 (5)	0.013 (6)	0.042 (6)	0.005 (4)	0.017 (5)	0.009 (4)
C1	0.029 (4)	0.014 (5)	0.031 (5)	0.007 (3)	0.011 (4)	0.009 (3)
C2	0.029 (4)	0.014 (5)	0.031 (5)	0.007 (3)	0.011 (4)	0.009 (3)
C3	0.052 (8)	0.022 (8)	0.038 (8)	0.021 (6)	0.014 (6)	0.002 (6)
C4	0.030 (6)	0.038 (9)	0.046 (8)	0.005 (6)	0.012 (6)	0.028 (7)
C5	0.050 (6)	0.027 (6)	0.044 (6)	0.005 (5)	0.012 (5)	0.018 (5)
C6	0.050 (6)	0.027 (6)	0.044 (6)	0.005 (5)	0.012 (5)	0.018 (5)
C7	0.029 (5)	0.024 (6)	0.042 (5)	0.013 (4)	0.018 (4)	0.011 (4)
C8	0.029 (5)	0.024 (6)	0.042 (5)	0.013 (4)	0.018 (4)	0.011 (4)

### Poly[[µ2-2,5,8,11,14,17-hexathia-[9.9](2,6,3,5)-pyrazinophane]tetra-µ-iodido-tetracopper(I)] (II) Geometric parameters (Å, º)

**Table:**

Cu1—N1	2.095 (10)	C1—C2^iii^	1.373 (17)
Cu1—S1	2.342 (4)	C1—C8	1.511 (18)
Cu1—S2	2.331 (4)	C2—C3	1.504 (19)
Cu1—I2^i^	2.5193 (18)	C3—H3A	0.9700
Cu2—S3	2.359 (4)	C3—H3B	0.9700
Cu2—I1	2.665 (2)	C4—C5	1.50 (2)
Cu2—I2	2.6166 (19)	C4—H4A	0.9700
I1—Cu2^ii^	2.675 (2)	C4—H4B	0.9700
Cu2—Cu2^ii^	2.663 (4)	C5—H5A	0.9700
S1—C3	1.803 (13)	C5—H5B	0.9700
S1—C4	1.834 (13)	C6—C7	1.50 (2)
S2—C5	1.779 (16)	C6—H6A	0.9700
S2—C6	1.808 (14)	C6—H6B	0.9700
S3—C7	1.793 (13)	C7—H7A	0.9700
S3—C8	1.803 (12)	C7—H7B	0.9700
N1—C1	1.346 (16)	C8—H8A	0.9700
N1—C2	1.364 (16)	C8—H8B	0.9700
			
N1—Cu1—S2	110.2 (3)	C2—C3—S1	116.7 (10)
N1—Cu1—S1	85.3 (3)	C2—C3—H3A	108.1
S2—Cu1—S1	91.74 (14)	S1—C3—H3A	108.1
N1—Cu1—I2^i^	121.1 (3)	C2—C3—H3B	108.1
S2—Cu1—I2^i^	112.48 (12)	S1—C3—H3B	108.1
S1—Cu1—I2^i^	130.61 (10)	H3A—C3—H3B	107.3
S3—Cu2—I2	109.54 (11)	C5—C4—S1	110.1 (10)
S3—Cu2—I1	105.86 (10)	C5—C4—H4A	109.6
I2—Cu2—I1	109.04 (8)	S1—C4—H4A	109.6
S3—Cu2—I1^ii^	99.22 (11)	C5—C4—H4B	109.6
I2—Cu2—I1^ii^	112.01 (7)	S1—C4—H4B	109.6
I1—Cu2—I1^ii^	120.18 (7)	H4A—C4—H4B	108.2
Cu2—I1—Cu2^ii^	59.82 (7)	C4—C5—S2	114.4 (10)
Cu1^iv^—I2—Cu2	94.93 (7)	C4—C5—H5A	108.7
Cu2^ii^—Cu2—I1	60.27 (7)	S2—C5—H5A	108.7
Cu2^ii^—Cu2—I1^ii^	59.91 (7)	C4—C5—H5B	108.7
S3—Cu2—Cu2^ii^	115.75 (13)	S2—C5—H5B	108.7
I2—Cu2—Cu2^ii^	134.68 (11)	H5A—C5—H5B	107.6
C3—S1—C4	100.9 (7)	C7—C6—S2	110.2 (10)
C3—S1—Cu1	96.4 (5)	C7—C6—H6A	109.6
C4—S1—Cu1	94.5 (5)	S2—C6—H6A	109.6
C5—S2—C6	104.7 (7)	C7—C6—H6B	109.6
C5—S2—Cu1	98.8 (5)	S2—C6—H6B	109.6
C6—S2—Cu1	105.0 (5)	H6A—C6—H6B	108.1
C7—S3—C8	102.8 (6)	C6—C7—S3	117.8 (10)
C7—S3—Cu2	106.0 (4)	C6—C7—H7A	107.9
C8—S3—Cu2	105.2 (5)	S3—C7—H7A	107.9
C1—N1—C2	116.6 (10)	C6—C7—H7B	107.9
C1—N1—Cu1	124.9 (9)	S3—C7—H7B	107.9
C2—N1—Cu1	118.5 (8)	H7A—C7—H7B	107.2
N1—C1—C2^iii^	122.1 (12)	C1—C8—S3	113.2 (9)
N1—C1—C8	117.1 (11)	C1—C8—H8A	108.9
C2^iii^—C1—C8	120.8 (11)	S3—C8—H8A	108.9
N1—C2—C1^iii^	121.3 (11)	C1—C8—H8B	108.9
N1—C2—C3	117.9 (11)	S3—C8—H8B	108.9
C1^iii^—C2—C3	120.7 (12)	H8A—C8—H8B	107.7
			
C2—N1—C1—C2^iii^	0.0 (19)	Cu1—S1—C4—C5	−52.9 (10)
Cu1—N1—C1—C2^iii^	179.6 (9)	S1—C4—C5—S2	62.8 (12)
C2—N1—C1—C8	177.4 (10)	C6—S2—C5—C4	74.9 (11)
Cu1—N1—C1—C8	−3.0 (15)	Cu1—S2—C5—C4	−33.3 (10)
C1—N1—C2—C1^iii^	0.0 (19)	C5—S2—C6—C7	−176.2 (10)
Cu1—N1—C2—C1^iii^	−179.6 (9)	Cu1—S2—C6—C7	−72.6 (10)
C1—N1—C2—C3	179.3 (11)	S2—C6—C7—S3	121.3 (9)
Cu1—N1—C2—C3	−0.3 (15)	C8—S3—C7—C6	−78.8 (11)
N1—C2—C3—S1	18.3 (17)	Cu2—S3—C7—C6	171.0 (9)
C1^iii^—C2—C3—S1	−162.3 (10)	N1—C1—C8—S3	−103.2 (11)
C4—S1—C3—C2	72.6 (13)	C2^iii^—C1—C8—S3	74.2 (14)
Cu1—S1—C3—C2	−23.2 (12)	C7—S3—C8—C1	96.8 (10)
C3—S1—C4—C5	−150.3 (10)	Cu2—S3—C8—C1	−152.4 (8)

Symmetry codes: (i) *x*+1, *y*, *z*; (ii) −*x*+1, −*y*+1, −*z*; (iii) −*x*+2, −*y*, −*z*; (iv) *x*−1, *y*, *z*.
